# Toxicity and Morphological Alterations Caused By Azadirachtin in the Stingless Bee *Nannotrigona testaceicornis* Lepeletier, 1836 (Insecta: Hymenoptera: Apidae: Meliponini

**DOI:** 10.1007/s13744-026-01421-7

**Published:** 2026-07-30

**Authors:** Andressa Santoro, Talita Aparecida Tódora Santana, Wingly Santos Beltrame, Nathalia Rodrigues da Silva de Carvalho, Larissa Eduarda de Oliveira Garcia, Fernanda Giovana Martins de Oliveira, Giovana Natiele Machado Esquissato, Vagner de Alencar Arnaut de Toledo, Daiani Rodrigues Moreira, Adriana Aparecida Sinópolis Gigliolli, Maria Claudia Colla Ruvolo Takasusuki

**Affiliations:** 1 Department of Biotechnology, Genetics, and Cell Biology, State University of Maringá, Maringá, Paraná, Brazil; 2 Department of Animal Science, State University of Maringá, Maringá, Paraná, Brazil

**Keywords:** Meliponini, Azadirachtin, Morphology, Bioinsecticide, CEC

## Abstract

*Nannotrigona testaceicornis* are essential pollinators for biodiversity maintenance and agricultural production. However, during foraging in agroecosystems, they may be exposed to residues of bioinsecticides used for pest management. Thus, this study evaluated the toxicity of the commercial product AzaMax® (azadirachtin) to the stingless bee *N. testaceicornis*. Adult worker bees were exposed to this bioinsecticide by contact and ingestion at concentrations recommended for strawberry crops, and mortality was assessed after 24, 48, and 72 h. Morphological alterations of the midgut and changes in chromatin structure in brain cells were evaluated in the surviving stingless bees. Exposure resulted in low mortality, precluding the estimation of the LC₅₀, but revealed significant sublethal effects, such as disorganization of the midgut epithelium, loss of digestive cells, reduction of regenerative cells, and disappearance of the peritrophic membrane. An increase in chromatin condensation was detected at different exposure times and concentrations, suggesting disruption of gene regulatory processes. These findings indicate that although azadirachtin does not induce immediate lethality in *N. testaceicornis*, it may compromise digestive physiology and genomic integrity, potentially affecting individual performance and long-term colony maintenance. The results highlight the importance of considering sublethal parameters in bioinsecticide risk assessments, as they may reveal hidden and ecologically relevant impacts that are not detected by mortality alone. By incorporating both lethal and sublethal endpoints, this research contributes to the development of safer agricultural practices and supports the conservation of stingless bees, which are key pollinators in tropical ecosystems.

## Introduction

Meliponines (Insecta: Hymenoptera: Apidae: Meliponini) are commonly known as stingless bees. Among them, *Nannotrigona testaceicornis* (Lepeletier, 1836) (Apidae: Meliponini), known in Brazil as Iraí, stands out. This species is widely distributed throughout Brazil and across Latin America and occurs in the Brazilian states of Bahia, Espírito Santo, Goiás, Mato Grosso do Sul, Minas Gerais, Paraná, Rio de Janeiro, Rio Grande do Sul, Santa Catarina, and São Paulo, as well as in Argentina (Misiones) and Paraguay (Camargo and Pedro 2013).

*Nannotrigona testaceicornis* is considered an important pollinator of crops such as melon, sunflower, and strawberry (Castilhos et al. 2019; A.B.E.L.H.A. 2015), playing an essential role in ecosystem maintenance and agricultural production and thereby contributing to global food security (Porto et al. 2021). However, during foraging in agricultural landscapes, these pollinators may come into contact with residues of synthetic pesticides through exposure to particles suspended in the atmosphere or deposited on plants, as well as through the ingestion of contaminated nectar and pollen (Krupke et al. 2012). In addition to impairing pollination services, intoxication may induce lethal and sublethal effects, including immune system impairment, behavioral alterations, and neurological and locomotor disorders (Desneux et al. 2007; Barbosa et al. 2015; Moreira et al. 2018; Bernardes et al. 2018).

In this context, bioinsecticides have been increasingly adopted as more sustainable alternatives to synthetic pesticides, with the aim of reducing the impact of chemical inputs on non-target organisms such as bees (Botti et al. 2015; Liu et al. 2021). Among these compounds, botanical insecticides that exhibit both repellent and insecticidal activity have been widely used in agriculture due to their rapid degradation, lower environmental persistence, and reduced impact on beneficial organisms, humans, and the environment (Miranda et al. 2016; Nicoletti et al. 2012).

Among botanical insecticides, azadirachtin, obtained from the seeds of the neem tree *Azadirachta indica* A. Juss., (Meliaceae), stands out (Campos et al. 2016; Sparks et al. 2001; Benelli et al. 2016). Azadirachtin is the active ingredient of commercial products such as AzaMax® (UPL), for which lethal and sublethal effects on pollinators, including stingless bees, have been reported, potentially compromising cellular integrity, behavior, and survival (Bernardes et al. 2017, 2018; Zhao et al. 2022).

Although most toxicological research has focused on *Apis mellifera* due to its economic importance (Arena and Sgolastra 2014), recent studies emphasize the urgent need to expand investigations on stingless bees. Species within the Meliponini clade exhibit distinct behaviors, physiology, and toxicological responses, and pesticide exposure has been shown to affect survival, cellular integrity, and social behavior in these species (Tomé et al. 2015; Boff et al. 2018; Toledo-Hernández et al. 2022; Oliveira et al. 2023).

Nevertheless, studies evaluating the effects of bioinsecticides on native Neotropical species such as *N. testaceicornis* remain scarce. This knowledge gap highlights the need for more comprehensive investigations to ensure the safety and conservation of these pollinators. Therefore, the aim of this study was to evaluate toxicity, as well as alterations in chromatin structure and midgut morphology, in adult worker stingless bees *N. testaceicornis* following exposure to the bioinsecticide AzaMax® (azadirachtin).

## Methods

### Biological Material

The experiments were conducted using adult forager workers of *Nannotrigona testaceicornis* collected from ten different colonies maintained in rational hives at the Experimental Farm of Iguatemi, State University of Maringá (UEM) (23° 82′ 5″ S, 51° 85′ 7″ W), located in the district of Iguatemi, Maringá, Paraná, Brazil. Worker bees were collected at the colony entrance as they returned from foraging. The colonies exhibited normal activity, with the presence of brood and food pots, and received supplemental syrup feeding twice a week. After collection, the bees were transported to the Laboratory of Biotechnology and Animal Genetics, Department of Biotechnology, Genetics and Cell Biology, State University of Maringá, Maringá, Paraná, Brazil (23° 25′ 30″ S, 51° 56′ 20″ W), where the experiments were conducted.

### Bioinsecticide

In this study, the insecticide AzaMax® was used; it is registered with the Brazilian Ministry of Agriculture, Livestock, and Food Supply (MAPA) under registration number 014807.

### Bioassays

The product was diluted according to the manufacturer’s recommendations for strawberry crops (3.0 g a.i./100 L). After preliminary mortality tests, the concentrations 2.25 × 10⁻^3^, 5.25 × 10⁻^3^, 7.5 × 10⁻^3^, 1.12 × 10^–3^, and 0.015 g a.i./mL were selected. Worker bees were then exposed to these concentrations by contact and oral exposure for 24, 48, and 72 h. For contact exposure, bees were placed in glass containers containing Candi food (a mixture of honey and powdered sugar) and filter paper (150 mm in diameter) soaked with 2 mL of the bioinsecticide solution at the different concentrations. The control group received the same Candi food and filter paper soaked only in water. The experiments were performed in triplicate, totaling three containers per treatment. Each container contained 15 worker bees, resulting in 45 bees per treatment. Considering the five tested concentrations and the control group (six treatments), a total of 270 bees were used throughout the experimental period (24, 48, and 72 h). For ingestion exposure, the filter paper was soaked with water, and the Candi food was contaminated with the predetermined concentrations. The control group received Candi food and filter paper soaked in water without bioinsecticide. The ingestion bioassay was conducted under the same experimental conditions, also using a total of 270 worker bees throughout the experimental period. Mortality was assessed after 24, 48, and 72 h of exposure, and the surviving bees from each evaluation period were subsequently used for the analyses of sublethal effects.

### Light Microscopy

After exposure to the bioinsecticide by ingestion at concentrations of 7.5 × 10⁻^3^, 1.12 × 10^–3^, and 0.015 g a.i./mL were selected for light microscopy analysis based on the mortality observed in the bioassay. For each experimental period (24, 48, and 72 h), four surviving bees per treatment were analyzed, totaling 16 bees per experimental period and 48 bees throughout the histological analysis. The bees were cold-anesthetized and dissected in physiological saline solution (0.1 M NaCl, 0.1 M Na₂HPO₄, and 0.1 M KH₂PO₄). The midguts were removed and fixed in aqueous Bouin’s solution. Samples were then dehydrated through a graded ethanol series, cleared in xylene, embedded in paraffin, and sectioned at 5 μm using a Leica RM 2250 microtome. Sections were stained with hematoxylin and eosin (H&E) (Junqueira and Junqueira 1983). Analyses were performed using an Olympus light microscope, and images were captured with a digital camera.

### Critical Electrolyte Concentration (CEC)

The critical electrolyte concentration (CEC) analysis was performed according to the protocol described by Mello et al. (1993). Following exposure to the bioinsecticide by contact, the concentrations of 7.5 × 10⁻^3^, 1.12 × 10⁻^3^, and 0.015 g a.i./mL were selected based on the mortality observed after 24, 48, and 72 h of exposure. For each experimental period, nine brains from surviving bees were analyzed per treatment, totaling 36 brains per experimental period and 108 brains throughout the entire analysis. The brains were dissected in physiological saline (0.1 M NaCl, 0.1 M Na₂HPO₄, and 0.1 M KH₂PO₄), spread onto microscope slides with 45% acetic acid, and, squashed under a coverslip. The slides were frozen in liquid nitrogen, and the coverslips were removed after returning to room temperature. The material was fixed in ethanol:acetic acid (3:1, v/v) for 1 min, and the slides were washed in ethanol for 3 min. Subsequently, the slides were stained for 20 min with 0.025% toluidine blue in McIlvaine buffer (pH 4.0) containing MgCl₂ at different concentrations (0.0, 0.02, 0.05, 0.08, 0.10, 0.12, 0.15, 0.20, and 0.30 mol/L). The slides were then washed in distilled water and stored. After drying, they were cleared in xylene for 15 min and mounted with Entellan for analysis under a light microscope. Cells with violet-stained nuclei were considered controls, whereas green coloration corresponded to the critical electrolyte concentration (CEC) point.

### Statistical Analysis

Normality and homogeneity of variances were assessed using the Kolmogorov–Smirnov, Shapiro–Wilk, and Bartlett tests, respectively. As these assumptions were not met (p < 0.05), nonparametric tests analyses were performed using the one-way ANOVA followed by Dunn (post hoc) test, with significance set at p < 0.05. Statistical analyses were conducted using GraphPad Prism 6 software.

## Results

### Mortality

**Fig. 1:**
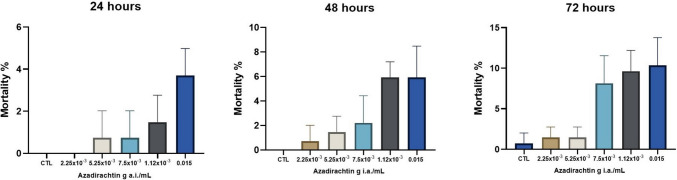
Mortality of *Nannotrigona testaceicornis* after exposure to different concentrations of AzaMax® by contact for 24, 48, and 72 h. Bars represent standard deviation

**Fig. 2:**
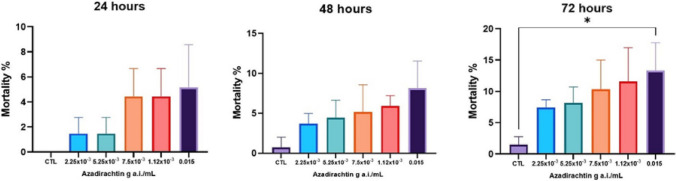
Mortality of *Nannotrigona testaceicornis* after exposure to different concentrations of AzaMax® by ingestion for 24, 48, and 72 h. Bars represent standard deviation. * indicates a significant difference (p-value < 0.05) according to the Kruskal–Wallis test

The different concentrations of azadirachtin did not significantly affect insect mortality following contact exposure at 24, 48, and 72 h (p > 0.05; Fig. 1). However, after ingestion, mortality differed significantly between the control group and the 0.015 g a.i./mL concentration at 72 h (Kruskal–Wallis χ^2^ = 10.18, df = 5, p = 0.0469; Fig. 2).

### Morphological alterations of the midgut of N. testaceicornis

**Fig. 3:**
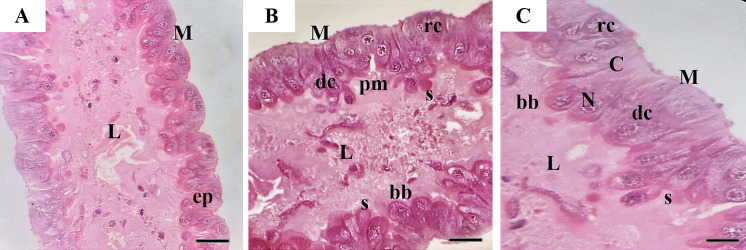
**A–C** Photomicrograph of the midgut of *Nannotrigona testaceicornis* without AzaMax® treatment. Muscle layers (M); epithelium (ep); digestive cells (dc); cytoplasm (C); nucleus (N); regenerative cell nests (rc); peritrophic membrane (pm); lumen (L); brush border (bb); secretion (s). Scale bars: A, B (100 μm); C (10 μm)

The midgut of the stingless bee *N. testaceicornis* is a cylindrical tube (Fig. 3A) lined by two muscle layers: an inner circular layer forming folds and an outer longitudinal layer (Fig. 3B, C). The lumen is delimited by the peritrophic membrane, which separates the luminal contents from the epithelium (Fig. 3B). The epithelium is composed of two cell types (Fig. 3B, C). Digestive cells are cylindrical, exhibit basophilic cytoplasm with the nucleus located in the median region, and are lined by a striated border in the apical region, where the release of part of the cytoplasm through secretory (apocrine) activity can be observed (Fig. 3B, C). Regenerative cells, in turn, occur in clusters (nests) located in the basal region of the epithelium (Fig. 3B, C). Each regenerative cell has a voluminous nucleus occupying most of the cytoplasmic volume.

**Fig. 4:**
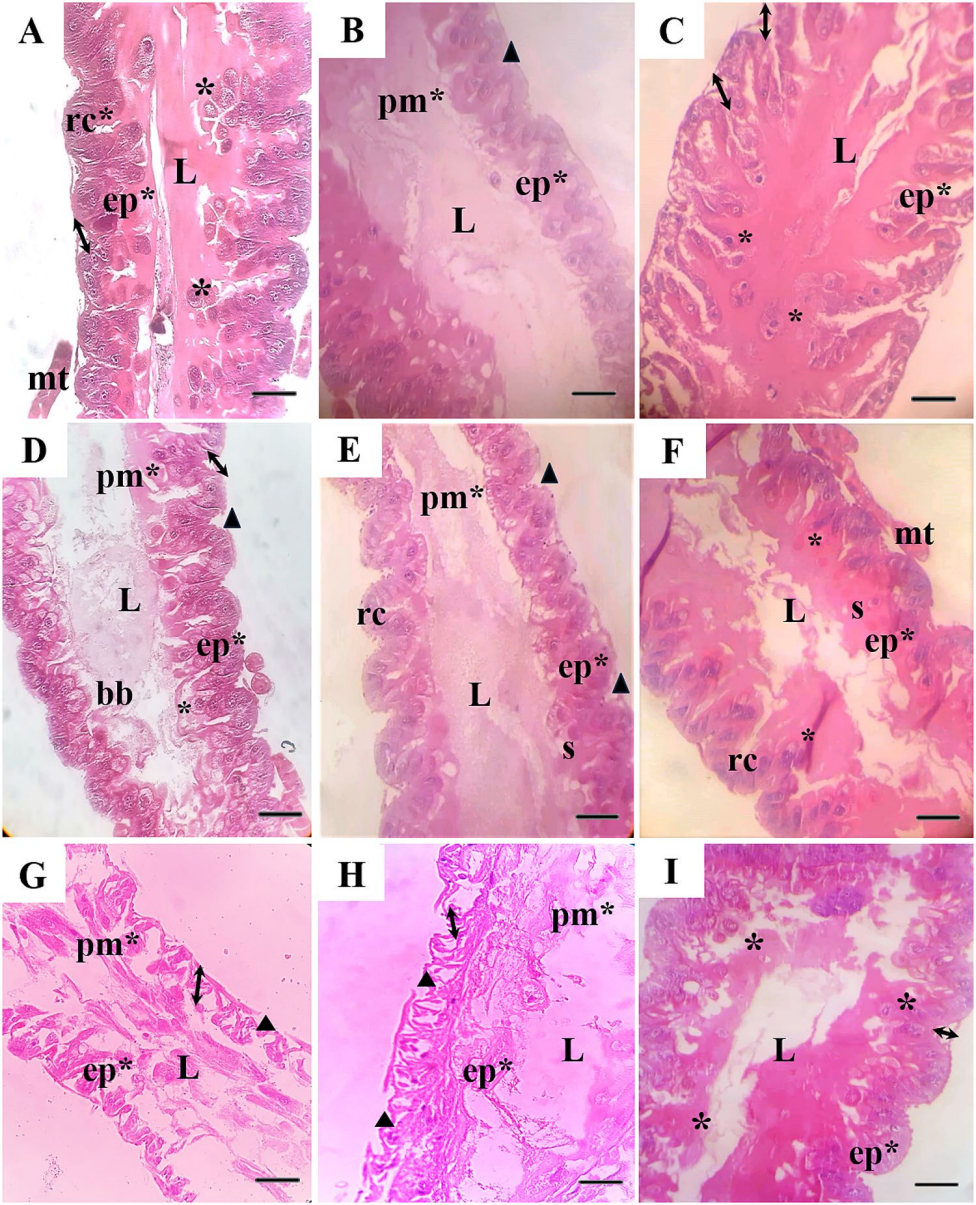
Photomicrographs of the midgut of *Nannotrigona testaceicornis* exposed by ingestion to AzaMax®. Treatments with 7.5 × 10⁻^3^ g a.i./mL (A–C) at 24 h (A), 48 h (B), and 72 h (C). Treatment with 1.12 × 10⁻.^3^ g a.i./mL (D–F) at 24 h (D), 48 h (E), and 72 h (F). Treatment with 0.015 g a.i./mL (G–I) at 24 h (G), 48 h (H), and 72 h (I). Regenerative cell nest (rc); brush border (bb); secretion (s); malpighian tubule (mt); absence of peritrophic membrane (pm*); degenerated epithelium (ep*); altered regenerative cell (rc*); muscle rupture (  ); detachment of the basal lamina from the muscle layer (  ) detachment and loss of digestive cells into the lumen (*). Scale bar: A–I (100 μm)

Bees exposed by ingestion to concentrations of 7.5 × 10⁻^3^, 1.12 × 10^–3^ and 0.015 g a.i./mL for 24 h exhibited midgut damage, including epithelial disorganization, detachment and loss of digestive cells into the lumen, and separation of the basal lamina from the muscle layer (Fig. 4A–C). With increased exposure periods of 48 and 72 h, the damage became more pronounced, characterized by the disappearance of the peritrophic membrane and regenerative cells, as well as further epithelial disorganization resulting from the loss of digestive cells into the lumen (Fig. 4D–I).

### Critical Electrolyte Concentration (CEC)

**Fig. 5:**
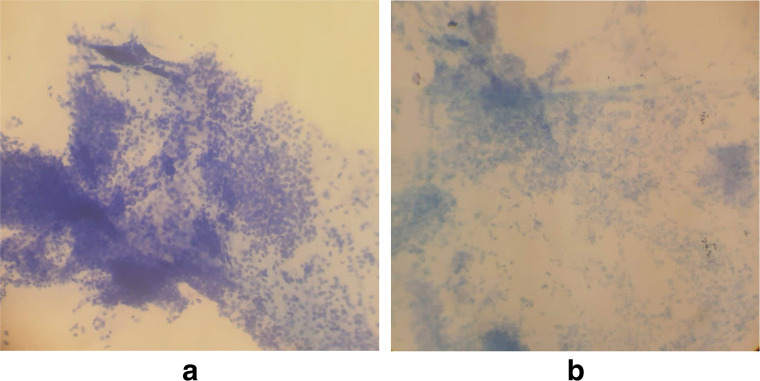
A–B. Nervous cells of *Nannotrigona testaceicornis* exposed to azadirachtin by contact and stained with toluidine blue in the presence and absence of magnesium chloride. (A) Control; (B) Critical electrolyte concentration (CEC) point. Light microscopy, 1000 × magnification

The CEC analysis showed that the control samples exhibited a CEC value of 0.15 M, indicating a lower degree of chromatin condensation (Fig. 5A). In contrast, the treated groups exhibited a higher degree of chromatin condensation (Fig. 5B).

For bees not exposed to the bioinsecticide, the critical electrolyte concentration (CEC) point remained constant at 0.15 M across all evaluated periods. After contact exposure to azadirachtin for 24 h, the CEC value was 0.15 M for the concentration of 7.5 × 10⁻^3^ g a.i./mL, 0.20 M for 1.12 × 10^–3^ g a.i./mL, and 0.30 M for 0.015 g a.i./mL. At 48 h, the CEC value was 0.30 M for all sublethal concentrations analyzed, indicating increased chromatin compaction. At 72 h, the CEC value was 0.30 M for the concentrations of 7.5 × 10⁻^3^ g a.i./mL and 0.015 g a.i./mL, and 0.20 M for the concentration of 1.12 × 10⁻^2^ g a.i./mL (Table 1).

**Table 1.**
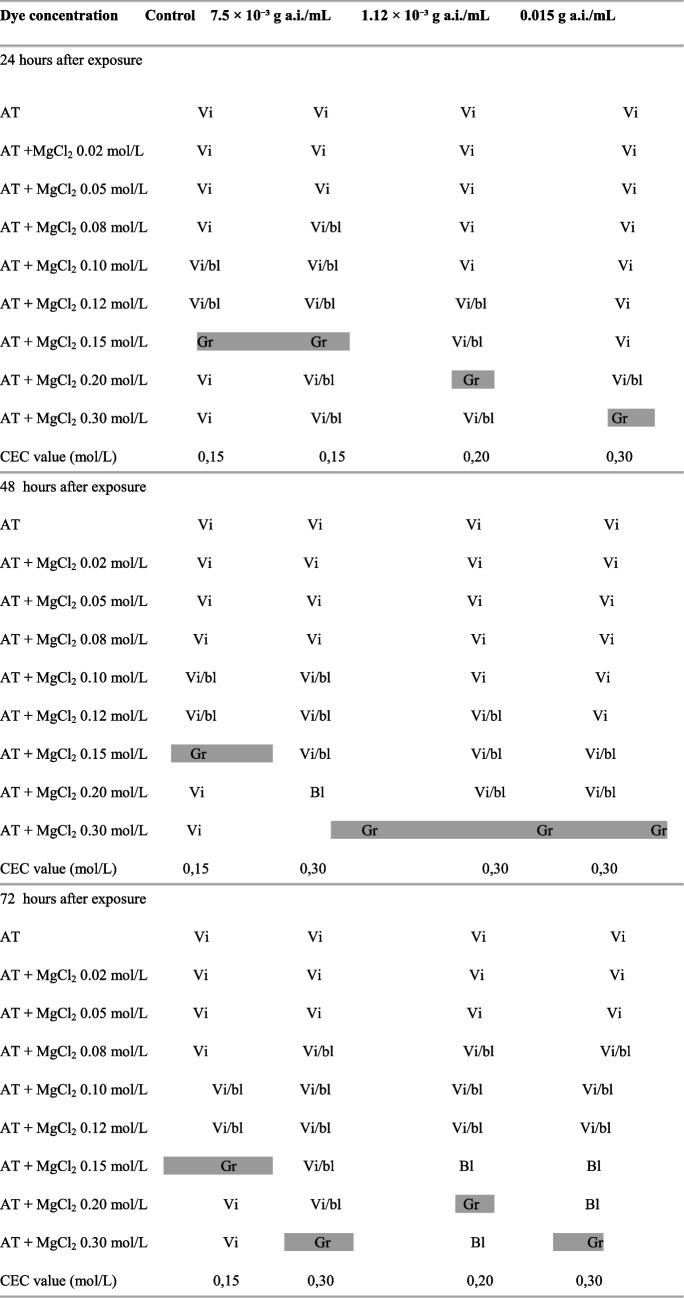
Nuclear chromatin basophilia responses in brain cells of *Nannotrigona testaceicornis* worker bees after 24, 48, and 72 h of oral exposure to the insecticide azadirachtin, stained with 0.025% toluidine blue (TB) supplemented with MgCl₂ at different concentrations

*Vi* violet, *Bl* blue, *Gr* green

## Discussion

Biopesticides are widely used as lower-risk alternatives to synthetic pesticides in integrated pest management programs, contributing to the reduction of negative effects on beneficial organisms (Barbosa et al. 2015). Among these products, those derived from *Azadirachta indica* stand out (Isman 2006; Pereira et al. 2018). However, when applied, residues may persist in agricultural environments, inadvertently exposing bees during foraging activities. Consequently, contaminated nectar and pollen may be collected and transported back to the colony, resulting in indirect intoxication of bees (Naumann et al. 1994; Naumann and Isman 1996; Efrom et al. 2012; Xavier et al. 2015).

The toxicity of bioinsecticides varies according to bee species, exposure time, and compound concentration (Lima et al. 2016). Nevertheless, several studies have reported adverse effects of azadirachtin on different species, including *Melipona scutellaris* (Neves et al. 2020), *Tetragonisca angustula* (Da Silva et al. 2024), *Apis mellifera* (Xavier et al. 2015; Amaral et al. 2015), *Trigona spinipes* (Correia-Oliveira et al. 2012), and *Tetragonula iridipennis* (Singh et al. 2015). This diversity of responses reinforces the need for species-specific approaches for meliponines, as physiological and metabolic differences among species may influence sensitivity to bioactive compounds.

In the present study, the exposure of *N. testaceicornis* worker bees to azadirachtin by ingestion and contact resulted in low mortality, a pattern similar to that observed in *Partamona helleri* (Bernardes et al. 2018), and for *Melipona quadrifasciata* and *P. helleri* 72 h after exposure to the bioinsecticide (Bernardes et al. 2017). Similarly, Wu et al. (2024) reported that azadirachtin was not acutely toxic to foraging bees but caused a significant inhibition of feeding. Although the acute toxicity of this compound appears limited for some bee species, chronic exposure to azadirachtin may induce sublethal effects that compromise colony maintenance.

Among the alterations observed, the damage to the midgut of *N. testaceicornis* was particularly noteworthy, as this organ represents one of the main interfaces between insects and the external environment. As the exposure period increased, progressive degeneration of the digestive cells and their release into the gut lumen were observed, demonstrating the degenerative effects of the bioinsecticide on the midgut tissue and impairing digestive and nutrient absorption processes (Cruz-Landim 2009). Similar alterations have been reported in *Apis mellifera* and *Partamona helleri* following exposure to the bioinsecticide spinosad, including disorganization of the midgut epithelium, a reduced number of regenerative cell nests, and the absence of the brush border in digestive cells (Lopes et al. 2017; Araujo et al. 2019). These alterations may impair midgut function, reducing the lifespan of bees as a consequence of compromised digestion and nutrient absorption (Oliveira et al. 2014).

Studies on bees exposed to pesticides have shown that intestinal alterations are associated with physiological impairments, including metabolic and genetic imbalances, oxidative stress, and changes in enzymatic activity, as well as behavioral alterations such as reduced foraging activity and impaired social behavior (Guedes et al. 2016, 2020; Bernardes et al. 2017; Oulhaci et al. 2018; Shimshoni et al. 2019; Shu et al. 2021; Zhao et al. 2022; Stuchi et al. 2023). For example, sublethal exposure to azadirachtin reduced the expression of immune-related genes and altered the activity of antioxidant enzymes in the midgut of *Apis cerana cerana* (Zhao et al. 2022). In *Apis mellifera*, a significant reduction in superoxide dismutase (SOD) activity, an enzyme essential for defense against reactive oxygen species, has been reported. This reduction may favor the accumulation of free radicals, leading to oxidative stress and cell death (Chakrabarti et al. 2020; López et al. 2007). In addition, an increase in glutathione S-transferase (GST) activity, an enzyme involved in xenobiotic detoxification, has also been reported (Habig et al. 1974; Papadopoulos et al. 2004; Yu et al. 1984). However, the intensity of insecticide-induced stress may exceed the cellular antioxidant and detoxification capacity, resulting in tissue damage and cell death. In *Apis mellifera*, exposure to the bioinsecticide spinosad impaired locomotor activity due to its neurotoxic effects (Araújo et al. 2023). Similar findings have also been reported for *Plebeia lucii* (Marques et al. 2020), indicating that reduced flight capacity may impair foraging activity, thereby compromising colony maintenance and reducing pollination services in agricultural ecosystems.

Another important effect observed in this study was chromatin condensation in brain cells of *N. testaceicornis* following exposure to azadirachtin. At 24, 48, and 72 h, exposed bees exhibited increased CEC values compared to the control group. A similar response was also observed in *Scaptotrigona bipunctata* following exposure to thiamethoxam (Moreira et al. 2018). Likewise, oral exposure of the same species to the fungicide Locker for 72 h resulted in an increased CEC value, indicating a higher degree of chromatin condensation (Diniz et al. 2020). Variations in CEC values are directly related to the structural state of chromatin: condensed chromatin exhibits higher CEC values than decondensed chromatin (Falco et al. 2010). This phenomenon occurs because, in condensed chromatin, toluidine blue molecules tend to stack, intensifying metachromasia and increasing the CEC value (Falco et al. 1999; Monteiro and Mello 1998). Moreover, chromatin condensation is associated with transcriptional repression and reduced gene activity, whereas decondensed chromatin corresponds to transcriptionally active states (Alberts et al. 2004).

In summary, although azadirachtin caused low mortality in *N. testaceicornis*, the sublethal effects observed in the midgut and brain chromatin reveal significant interference with worker physiology, behavior, and gene regulation. These findings reinforce the notion that bioinsecticide risk assessment should extend beyond immediate lethality and incorporate analyses capable of detecting subtle yet potentially persistent impacts on individual and colony health. Considering the essential role of stingless bees in maintaining tropical ecosystems and supporting agricultural sustainability, these results underscore the need for caution in the use of plant protection products, even those commonly classified as “natural” or low-impact.

## Data Availability

All data is available upon request to the corresponding author.
